# In-silico prediction of multi‑target mechanisms of Pinellia ternata phytochemicals in lung cancer: Evidence from a graph‑attention‑guided virtual screening and multi‑scale simulations

**DOI:** 10.1371/journal.pone.0349376

**Published:** 2026-05-18

**Authors:** Guoqiang Bian, Yuanbin Zhang, Yuanhao Shen, Pengcheng Xiao, Daifeng Zhang, Jiadong Xie, Xiong Li, Duo Chen, Kongfa Hu, Chenjun Hu

**Affiliations:** 1 School of Artificial Intelligence and Information Technology, Nanjing University of Chinese Medicine, Nanjing, China; 2 Jiangsu Collaborative Innovation Center of Traditional Chinese Medicine in Prevention and Treatment of Tumor, Nanjing, China; 3 Jiangsu Province Engineering Research Center of TCM Intelligence Health Service, Nanjing University of Chinese Medicine, Nanjing, China; University of Botswana, BOTSWANA

## Abstract

Pinellia ternate has long been used to treat respiratory diseases, possessing potential anti-tumor activity and exhibiting multi-component, multi-target characteristics. This study prioritized lung cancer-related targets using the HERBGAT framework based on graph attention networks (GAT). High-quality PDB structures were retrieved, and diffusion-generative docking was performed to construct complex conformations and assess their confidence levels. Molecular dynamics simulations of representative complexes were conducted over 200 ns, and binding free energies were estimated using the MM/PBSA method. The pharmacokinetic characteristics of the bioactive compounds were evaluated using Swiss ADME and PreADMET computational tools, and density functional theory (DFT) analysis using ORCA software was combined to explore their electronic structure and properties. In this study, the potential targets of Pinellia ternata highly overlap with lung cancer pathological genes, with FGFR4, CDK2, JAK2, KDR, PAK4, PTK2 and PDGFRA being the core. Baicalein exhibits a conserved binding mode of “hinge hydrogen bond-aromatic interlayer-hydrophobic groove” at targets such as PTK2/KDR/JAK2. Energy decomposition indicates that van der Waals forces and nonpolar solvation are the main thermodynamic driving forces for complex formation. Density functional theory (DFT) analysis further reveals that the high electronic “softness” of baicalein and its sensitive response to the environment in terms of frontier orbitals and electrostatic potential may be related to its high affinity, which is ubiquitous in different pockets. This study provides a computational chain of evidence for the intervention of Pinellia ternata’s active ingredient on lung cancer-related targets. The well-defined cross-target migratory pharmacophore of baicalein, consistent energy and kinetics, and the oral pharmacodynamics of ADMET indicate that it can serve as a multi-target lead compound targeting the PTK2/KDR migration-angiogenesis pathway, while also affecting JAK2 and CDK2. Given that the current evidence is based on in-silico predictions, further validation through target enzymology, binding thermodynamics, and cellular pathway experiments is needed.

## Introduction

Lung cancer remains the leading cause of cancer-related deaths worldwide, with its high mortality rate primarily due to diagnostic delays and the limited efficacy and tolerability of late-stage treatments [1]. Although targeted therapy and immunotherapy have significantly improved the prognosis for some patients, the prevalenc e of acquired drug resistance and toxic reactions underscores the urgent need to develop novel intervention strategies with lower toxicity and broader effects. In this context, natural products and traditional ethnic medicines, with their multi-component and multi-target features, are considered an important complement to modern single-target drugs; among them, traditional Chinese medicine offers a unique approach to the systemic intervention of complex diseases through the holistic regulation of multiple pathological pathways in synergistic compounds.

Pinellia ternata has been highly regarded in traditional medicinal formulas for generations, used to expel phlegm, stop coughs, alleviate asthma, and other respiratory symptoms. It is also often used as a component of adjuvant therapy for cancer patients. Previous reports suggest that preparations related to Pinellia ternata can affect key pathways such as PI3K/Akt [2], demonstrating potential anti-tumor activity. However, the specific molecular targets and mechanisms by which Pinellia ternata exerts its anti-lung cancer effects are still not systematically elucidated. The integration of modern systems pharmacology and computational biology provides a feasible path for deciphering the multi-layered association of “herb-component-target-pathway”, especially network analysis based on graph representation learning [3], which can capture potential herb-disease-gene coupling relationships in heterogeneous biological networks. Network pharmacology which based on systems biology and centered on multi-target and multi-pathway approaches, emphasizes explaining and utilizing the overall regulatory potential of multi-component natural products in complex diseases by starting from a multi-level map of compound-target-pathway-phenotype. Drug efficacy is not determined solely by individual high-affinity nodes, but rather than shaped by the relationships of multiple nodes and edges. In the context of tumor immunology, an integrated “computational evidence chain” has recently emerged as a consensus [4]. In the direction of immune checkpoints, natural small molecules have been shown to form a conserved pharmacophore across targets, involving “hinge hydrogen bonds-aromatic gating-hydrophobic grooves”, thus exhibiting stable binding at different receptor sites primarily driven by ΔEvdW. The consistency of molecular docking, MD simulations, energetics, and bioinformatics provides important support for preliminary validation [5,6].

The study bases on the frameworks of network pharmacology and graph attention networks, pick up disease-associated genes for lung cancer from databases including TCGA [7], HGNC [8], OMIM, STRING and GeneCards. And then construct a complex network connecting traditional Chinese medicine(TCM) [9], genes, and proteins, perform mining through this network and output potential TCM-associated genes. We search the active components of Pinellia ternata from the TCMSP database. Screening based on oral bioavailability and drug likeness yielded a set of natural compounds with development potential. This study focuses on 12 compounds in Pinellia ternata: baicalein, Baicalin, Xanthosine, (-)-beta-Sitosterol, Cavidine, coniferin, Cycloartenol, cis-11-Eicosenoic acid, Stigmasterol, 10,13-Eicosadienoic acid, 12,13-Epoxy-9-hydroxynonadeca-7,10-dienoic acid and 24-Ethylcholest-4-en-3-one. Studies have shown that baicalin exerts antitumor activity through multiple pathways, such as inhibiting angiogenesis and cell proliferation. While phenylpropanoid glycosides typically exhibit antioxidant and antiproliferative potential; and nucleoside compounds can affect rapidly dividing cells by interfering with nucleotide metabolism and signal transduction. These compounds complement each other in terms of skeleton, polarity, and polarizability, forming a chemical basis for potential synergistic effects.

**Fig 1:**
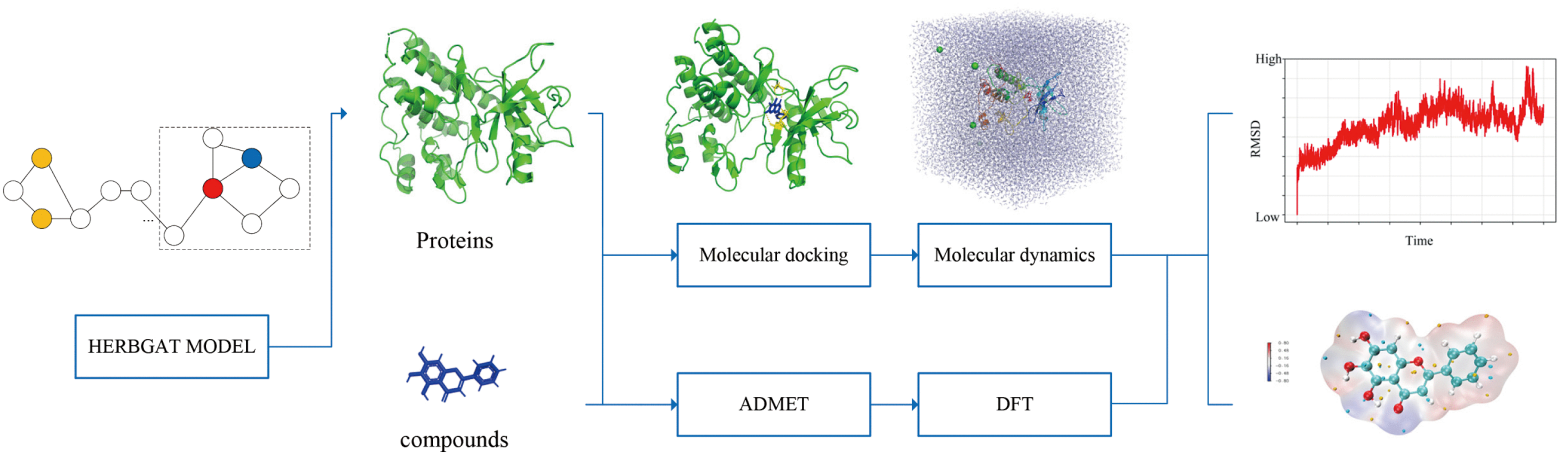
The process of CrossScale‑Herb.

In methodology, we constructed a hierarchical integrated computational evidence chain which named CrossScale-Herb as is shown in Fig 1, utilizing virtual screening and molecular docking to obtain the initial binding posture, verifying the stability of the complex in the aqueous solution system through molecular dynamics, and estimating the relative binding free energy with MM/PBSA. Combining in vivo and in vitro ADMET exploitability prediction [10] and DFT electronic structure analysis, we systematically evaluated the possibility and rationale for the key components of Pinellia ternata to act on lung cancer-related targets, especially CDK2/JAK2/KDR/PAK4/PTK2 etc.

## Materials and methods

### Ethics statement

This study did not involve interaction with human participants or access to identifiable private information. All analyses were performed on publicly available, de-identified datasets, including TCGA (accessed the GDC on 1 May 2024; project(s): TCGA-LUAD/LUSC; only open-access data were used), HGNC (accessed on 1 May 2024), OMIM (accessed on 15 May 2024), STRING (accessed on 30 May 2024), and GeneCards (accessed on 30 June 2024). According to the policies and applicable regulations, the use of publicly available de-identified data does not constitute human subjects research and does not require IRB review; therefore, informed consent was not required. The authors had no access to information that could identify individual participants during or after data collection. The study posed no risks to individual privacy, and all repository terms of use were followed.

### Network pharmacology target identification

**Fig 2:**
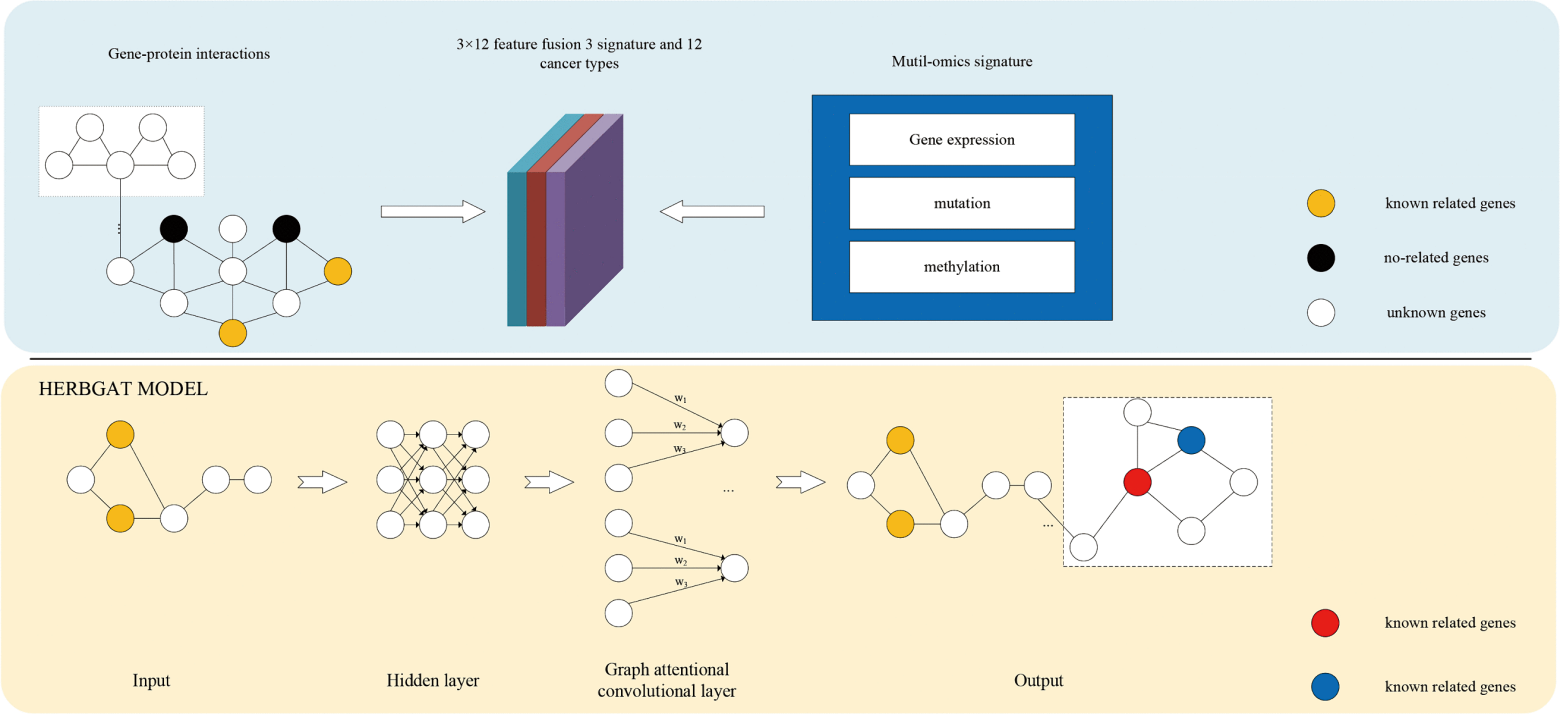
HERBGAT overall architecture.

The starting point for this study was a gene set containing potential target genes associated with Pinellia ternata treatment for lung cancer. This gene set was constructed based on the HERBGAT [11] model, the architecture of which is shown in Fig 2. In previous studies, researchers used the HERBGAT graph neural network model with an attention mechanism and the improved EMOGI algorithm to predict genes associated with Pinellia ternata and lung cancer, respectively, and obtained the high-confidence target list by taking the intersection of the two. Given that previous studies have verified the biological relevance of this gene set through bioinformatics analysis, this study used it as an unscreened comprehensive target set, aiming to systematically evaluate the potential binding ability of key active components of Pinellia ternata to various proteins in this target set through large-scale virtual screening.

### Protein structure and compounds identification

The Pinellia ternata compounds were obtained from the TCMSP database, which initially contained 116 phytochemical components. The 12 compounds ultimately selected were obtained through a rigorous pharmacokinetic screening process, using standard thresholds of oral bioavailability (OB≥30%) and drug-likeness (DL≥0.18) to exclude candidate compounds with poor pharmacological properties. Structural data were obtained from the RCSB Protein Database (PDB). We prioritized high-resolution (≤3.0 Å) structures of target genes resolved by X-ray crystallography or cryo-electron microscopy (cryo-EM). When multiple PDB entries existed for the same gene, we prioritized structures co-crystallized with small molecule ligands to accurately define the active pocket. Proteins with only key functional domains resolved (e.g., kinase domains) were also included in our analysis. All acquired PDB structures were filtered using a text pattern matching-based filter to remove all non-canonical biopolymer entities from the PDB files, resulting in a clean set of protein atomic coordinates. These structures were then submitted to the PDBFixer module for structural integrity repair and normalization. Finally, we optimized the hydrogen bond network using the Reduce module. Simultaneously, using the Traditional Chinese Medicine Systems Pharmacology Database (TCMSP) and PubChem as primary data sources, we applied a pharmacokinetic-based screening method, i.e., oral bioavailability (OB)≥30% and drug-likeness (DL)≥0.18, to enrich molecules with potential in vivo activity. This screening strategy ultimately yielded 12 candidate compounds(S1 Fig). For the few targets in the PDB that lacked experimental structures or had poor structural quality, we considered using the AlphaFold3 prediction model [12] as a supplement. However, preliminary screening revealed that the targets most strongly associated with Pinellia ternata-lung cancer, such as FGFR4, CDK2, BTK, AR, and PDGFRA, all possessed high-quality crystal structures. This allowed our high-throughput molecular docking studies to be primarily based on experimentally resolved structures, thus ensuring the reliability of the receptor conformation.

### Diffusion-based molecular docking

This study defines molecular docking as a generative modeling task to explore the interaction patterns between active compounds of Pinellia ternata and lung cancer-related target proteins. The study employs the DiffDock [13] method, which generates precise binding postures of small molecules and target proteins based on a diffusion model, rather than relying on traditional search paradigms that optimize scoring functions. This avoids deviations between predicted results and actual physical conditions caused by model uncertainties. The core of DiffDock is a diffusion-generative model defined on the ligand posture manifold. To ensure consistency between the posterior distribution of the final combined postures and the model’s learning results, the model iteratively “denoises” the prior distribution of information loss through a reversible stochastic process and progressively optimizes the training of the translational, rotational, and torsional degrees of freedom of the ligands. For each target protein, the binding pocket is defined based on its crystal structure and the resolved cocrystal ligands. If prior information is lacking, the surface cavity with the most significant geometric meaning identified by the CASTp server is used as a substitute. The docking computation process comprises two main stages: First, DiffDock’s generative model simultaneously generates dozens of candidate conformations for each compound-protein pair. Then, a separately trained confidence model evaluates and ranks these generated conformations. This confidence model aims to predict the probability that a given conformation has a root mean square deviation (RMSD) of less than 2 Å from the native structure. The confidence score can be used to screen for high-quality binding modes.

### Molecular dynamics simulations

Simulations were performed using GROMACSv2024 (http://www.mdtutorials.com/gmx/complex/index.html) and an AMBER force field [14]. The system was subjected to three-dimensional periodic boundary conditions (PBC). First, energy minimization was performed using the steepest descent method [15]. Then, the system was equilibrated under NVT and NPT ensembles, respectively. A 100 ps constant isochoric equilibrium (NVT) was performed at a steady-state temperature of 300 K using the Berendsen thermostat algorithm with a coupling constant of 0.1 ps. Subsequently, a 1 ns constant pressure equilibrium (NPT) was performed at 1 bar using a Parrinello-Rahman barostat with a coupling constant of 5.0 ps. The range of interactions was calculated using the particle mesh Ewald (PME) method. The calculated cutoff radius for long-range electrostatic interactions and short-range nonbonding interactions was 12 Å. The simulation was repeated twice, each lasting 200 ns. Molecular dynamics trajectory analysis was used to calculate the root mean square deviation (RMSD), root mean square fluctuation (RMSF), radius of gyration (Rg), number of hydrogen bonds, and principal component analysis (PCA) [16].

### MM-PBSA calculations

The total binding free energy between ligand and protein is measured by combining molecular dynamics simulations and thermodynamic techniques (mechanical/Poisson-Boltzmann surface area (MM-PBSA) method) [17]:


$$\begin{array}{c} \Delta G_{binding}=\Delta G_{MM}+\Delta G_{sol}-T\Delta S \end{array}$$ (1)

where G(complex) is the total free energy of the protein-ligand complex, and G(receptor) and G(ligand) are the free energies of the isolated protein and ligand in the solvent, respectively. The total free energy of the three entities (complex, receptor, or ligand) can be calculated by adding their molecular mechanical potential ($\Delta {\text{G}}_{MM}$) to their solvation energy ($\Delta {\text{G}}_{sol}$) [18]. Intermolecular van der Waals forces ($\Delta {\text{E}}_{vdW}$), electrostatic interactions ($\Delta {\text{E}}_{elec}$), and nonpolar solvation energy ($\Delta {\text{E}}_{np}$) all favor binding. However, polar solvation free energy ($\Delta {\text{E}}_{pol}$) and configurational entropy (-TΔS) are unfavorable for ligand-protein binding. Therefore, the total free binding energy of the ligand-protein complex was calculated using the MM/PBSA GROMACS program via MD trajectory [19].

### ADMET profile

The physicochemical and pharmacokinetic properties, including absorption, distribution, metabolism, excretion, and toxicity, of selected baicalein, Baicalin, Xanthosine, (-)-beta-Sitosterol, Cavidine, coniferin, Cycloartenol, cis-11-Eicosenoic acid, Stigmasterol, 10,13-Eicosadienoic acid, 12,13-Epoxy-9-hydroxynonadeca-7,10-dienoic acid and 24-Ethylcholest-4-en-3-one were investigated. Results were obtained through the Swiss ADME and pre-ADME online servers (http://preadmet.bmdrc.org/).

### DFT study

Density functional theory (DFT) analysis of the active components of Pinellia ternata was performed using ORCA 5.0.1 [20–33], and visualization was performed using Multiwfn [34,35] and VMD [36]. The structural coordinates of the selected compounds were optimized using the B3LYP-D3/def2-TZVP basis set without imposing any symmetry constraints. From the optimized geometry, the optimized surface potential, HOMO-LUMO energy levels, and thermochemical parameters (thermal energy, enthalpy, Gibbs free energy, thermal entropy, hardness, softness, and electron affinity) of baicalein, Baicalin, Xanthosine, (-)-beta-Sitosterol, Cavidine, coniferin, Cycloartenol, cis-11-Eicosenoic acid, Stigmasterol, 10,13-Eicosadienoic acid, 12,13-Epoxy-9-hydroxynonadeca-7,10-dienoic acid and 24-Ethylcholest-4-en-3-one were obtained.

## Results and discussion

### Herbal-disease target prediction

Network pharmacology analysis shows a significant overlap between the potential targets of Pinellia ternata and the pathological gene network of lung cancer. Among the top 500 genes in both groups, we identified common molecular targets. This high degree of overlap strongly suggests that the active components of Pinellia ternata can broadly intervene in key signaling pathways in lung cancer. Further analysis of these co-occurring targets indicates that most are oncogenes, tumor suppressor genes, and signaling kinases closely related to tumorigenesis and development. Among them, targets such as FGFR4 [37,38], CDK2 [39,40], JAK2 [41,42], KDR [43,44], PAK4 [45,46], PTK2 [47,48] and PDGFRA [49,50] are particularly prominent because they play crucial roles in core cancer processes such as cell proliferation, cell cycle regulation, tumor microenvironment and angiogenesis which is strongly associated with tumors as lung cancer and colorectal cancer. Furthermore, we mapped the identified disease targets to the STRING database (v12.0). Under the conditions of setting the species to Homo sapiens, minimum required interaction score > 0.7, and including all evidence sources, we constructed a high-confidence PPI network. This network was then imported into Cytoscape v3.10.4 for visualization and reconstruction, and integrated with TCMSP and KEGG data to construct a component-target-pathway network (S5 and S6 Figs). Based on the PPI network, we used the CytoHubba plugin to analyze the network topology, and employed the MCC (Maximal Clique Centrality) algorithm as the core evaluation metric to screen and identify key hub nodes (S7 Fig).

### Molecular docking studies

**Fig 3:**
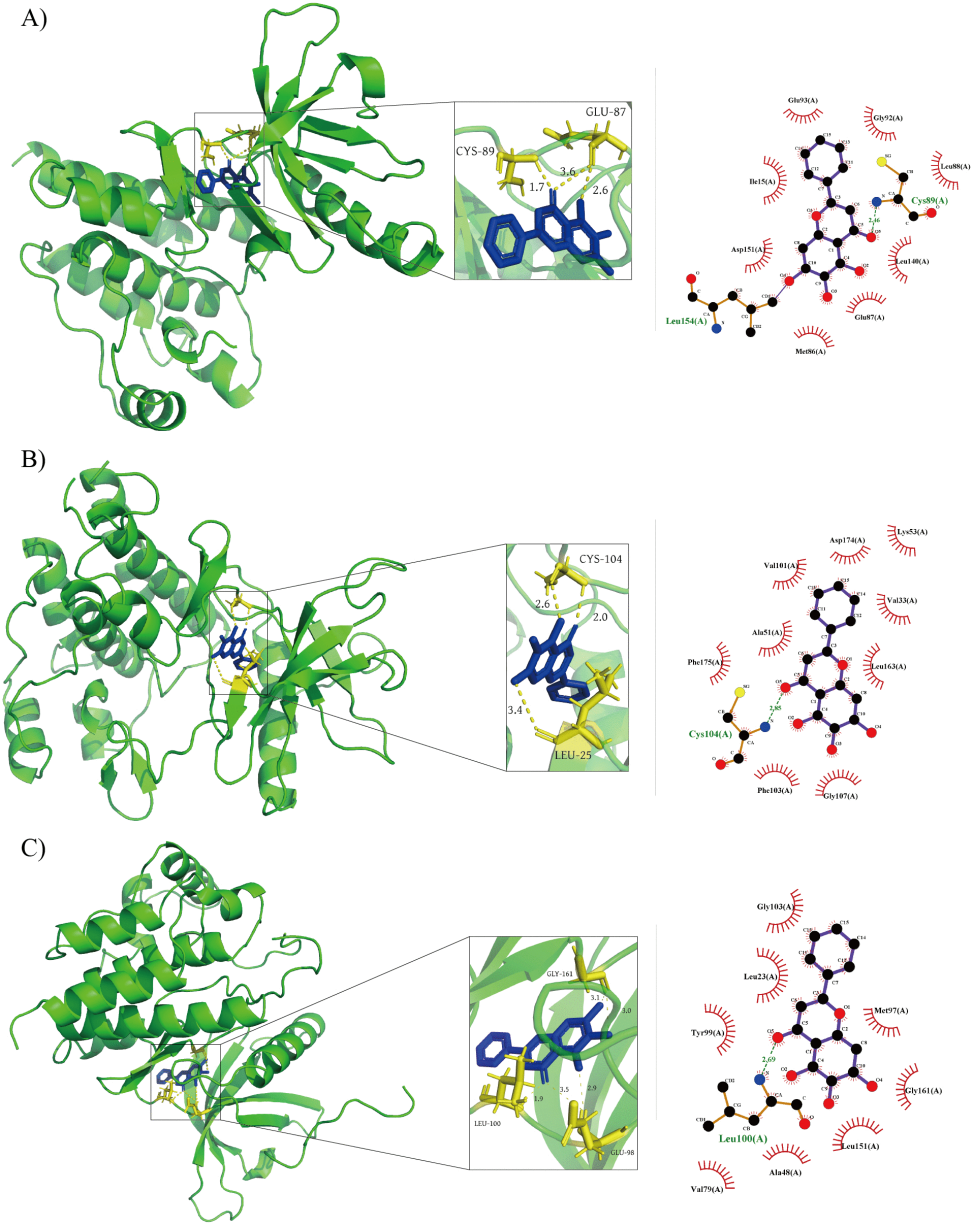
Interaction and orientation of baicalein in A) PTK2, B) KDR, and C) JAK2.

We used DiffDock to dock the active ingredient of Pinellia ternata with target proteins. The resulting model generated a set of energy-favorable conformations for each ligand-protein complex(S1 Table). We selected the optimal binding posture based on a docking scoring function for further interaction analysis(S3 Fig). From the docking results, we found that the docking confidence of baicalein with some lung cancer-related genes was high(S1 File). Among them, the binding states of baicalein with PTK2 and KDR with JAK2 were the most stable at the lung cancer-related gene targets. The three-dimensional and two-dimensional complexes and internal interactions of these three complexes are shown in Fig 3. PTK2 dominates the adhesion-migration-invasion axis and is an important node in lung cancer metastasis and colorectal cancer invasion. In the PTK2 complex, baicalein forms a strong anchorage with the NH of the Cys89 backbone in the hinge region (H-O approximately 2.46 Å), and the adjacent Glu87 provides a short-range polar boost in the range of 2.6–3.6 Å, forming a two-point fixed posture. Meanwhile, the aromatic plane of baicalein is enclosed by Leu88, Leu140, Ile15, and Met86, and the two-dimensional plot also shows the additional hydrophobic attachment of Leu154, which constitutes a well-solvent-shielded microcavity. This geometry reduces the cost of desolvation and reduces conformational entropy loss through the π−π/CH-π network. The function of KDR in mediating tumor angiogenesis can serve as one of the focal points for anti-metastasis and anti-angiogenesis. The complex of baicalein and KDR exhibits a Type I adenine pocket-occupancy mode. We found that the carbonyl group of baicalein forms a bond of about 2.85 Å with the NH group of the hinge Cys104 backbone, and the distal aromatic core forms a stable π−π stack with Phe175. Val101, Ala51, Leu163, and Val33 form a continuous hydrophobic trench in the lateral direction. The density of the contact network and the geometrical coaxiality of the aromatic interlayer indicate significant contributions from van der Waals forces and dispersion in the binding interaction. Although the DFG remains in the “in” state and the post-cavity is not fully utilized, the matching of the anterior cavity-gatekeeper channel is sufficient to support high-quality docking. JAK2 is highly correlated with the JAK/STAT signal axis in the inflammation-survival-proliferation circuit. Its corresponding complex is based on the 2.69 Å hydrogen bonds of the NH group in the Leu100 backbone. The gatekeeper region Met97/Tyr99 forms a tight aromatic interlayer with the ligand. Leu23, Ala48, Val79, and Leu151 transform the channel into a narrow cavity, thus maintaining a high-affinity, hydrophobic-π-interaction-dominated binding posture even in the absence of significant salt bridges. Based on this, this study prioritizes PTK2 and KDR as the main targets in lung cancer-related targets, with JAK2 as a key auxiliary target. Baicalein shares a conserved binding core of “hinge receptor hydrogen bond and gatekeeper aromatic interlayer” across these three targets, while also gaining potential selective space through differences in lateral cavity geometry. Subsequently, molecular dynamics and free energy perturbation can be carried out on the basis of these three complexes to verify the residence time of hinge hydrogen bonds and the degree of water network substitution. Then, site-directed modification can be carried out based on the receptivity of the gatekeeper lateral cavity to complete the lead optimization from multi-target framework to disease pathway guidance.

### Molecular dynamics simulation results

**Fig 4:**
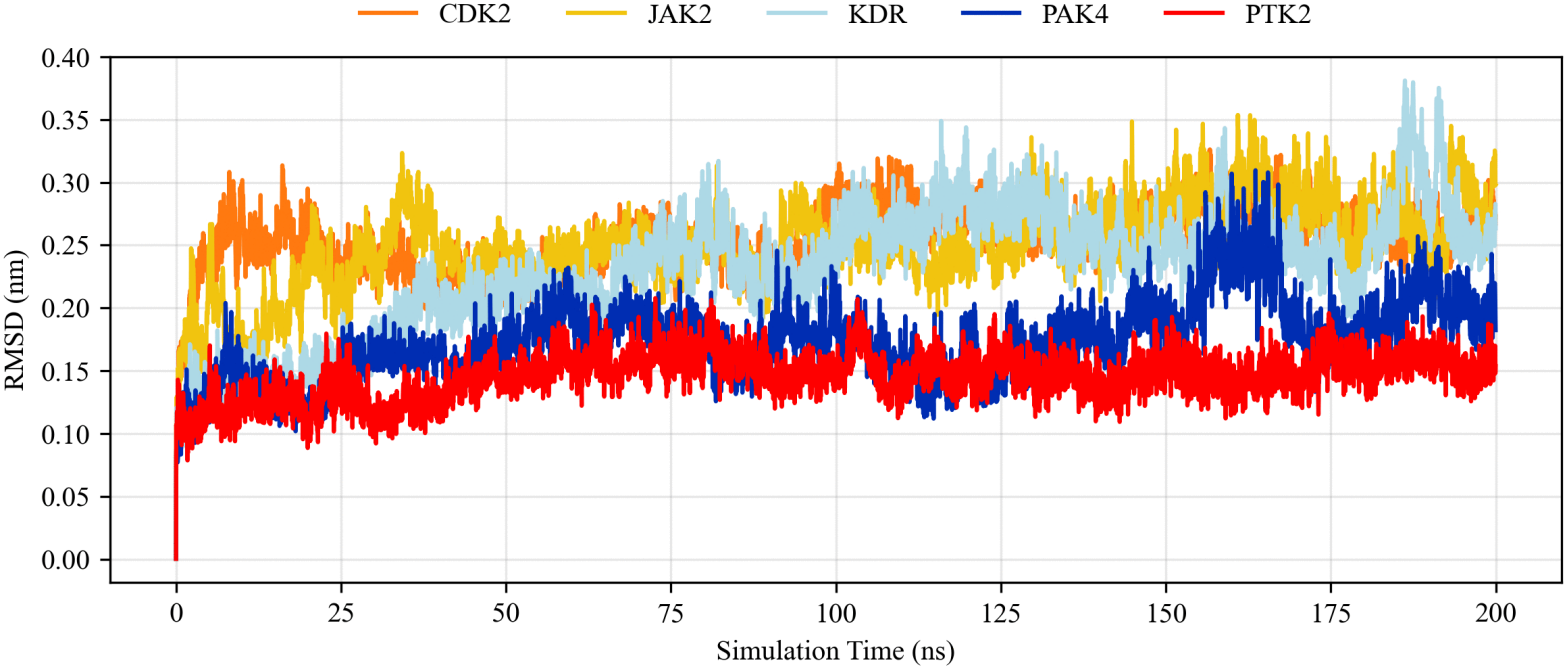
RMSD backbones of baicalein in complex with different receptor proteins.

We used molecular dynamics simulations to investigate the stability of molecular docking complexes [45] under dynamic conditions. Molecular dynamics simulations were performed on selected protein-ligand complexes over a period of 200 ns. Trajectory analysis showed that most complexes were indeed stable, with ligands remaining bound and key interactions preserved. We focused on the docking complexes of top-ranked baicalein with CDK2, JAK2, KDR, PAK4, and PTK2. Fig 4 shows the root mean square deviation (RMSD), root mean square smoothness coefficient (RMSF), relative molecular mass (Rg), and number of hydrogen bonds. RMSD calculations were used to assess conformational fluctuations of the protein-ligand backbone atoms and the stability of the simulated systems over a 200 ns simulation period [51]. As shown in Fig 4, all systems reached a final stable state after experiencing initial minor fluctuations caused by dynamic shocks. Almost all systems experienced initial dynamic shocks during molecular dynamics studies. The RMSD results indicate that the selected ligands reached a stable level in all systems, demonstrating consistent stability of the ligands at the protein active sites. Therefore, it can be concluded that the ligand-protein complex did not cause any significant conformational changes in the protein structure.

**Fig 5:**
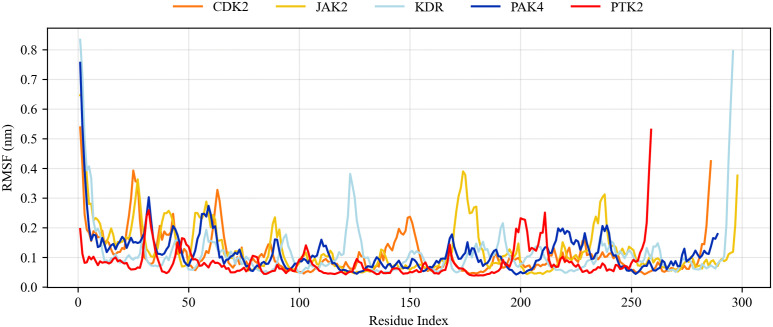
RMSF plots of baicalein in complexes with different receptor proteins.

RMSF analysis determines the variation of amino acid residues by calculating the average value of amino acid residue atoms [52]. The smaller the RMSF value, the smaller the conformational change of the protein-ligand complex. Fig 5 shows the RMSF results of baicalein complexes with corresponding proteins. The RMSF of all amino acid residues in the receptor active site does not exceed 0.4 nm. It is expected that the variation amplitude of the loop region, C-terminus, and N-terminus regions will be greater than that of the residues at the binding site and will participate in ligand-protein interactions. The RMSF plots of unbound and bound proteins confirm that the binding of the ligand to the protein does not change the protein structure [53]. In addition, the residual variation is less than 0.4 nm, indicating that the ligand-protein complex has no significant effect on the protein backbone, which is consistent with the RMSD results.RMSF analysis determines the variation of amino acid residues by calculating the average value of amino acid residue atoms [52]. The smaller the RMSF value, the smaller the conformational change of the protein-ligand complex. Fig 5 shows the RMSF results of baicalein complexes with corresponding proteins. The RMSF of all amino acid residues in the receptor active site does not exceed 0.4 nm. It is expected that the variation amplitude of the loop region, C-terminus, and N-terminus regions will be greater than that of the residues at the binding site and will participate in ligand-protein interactions. The RMSF plots of unbound and bound proteins confirm that the binding of the ligand to the protein does not change the protein structure [53]. In addition, the residual variation is less than 0.4 nm, indicating that the ligand-protein complex has no significant effect on the protein backbone, which is consistent with the RMSD results.

**Fig 6:**
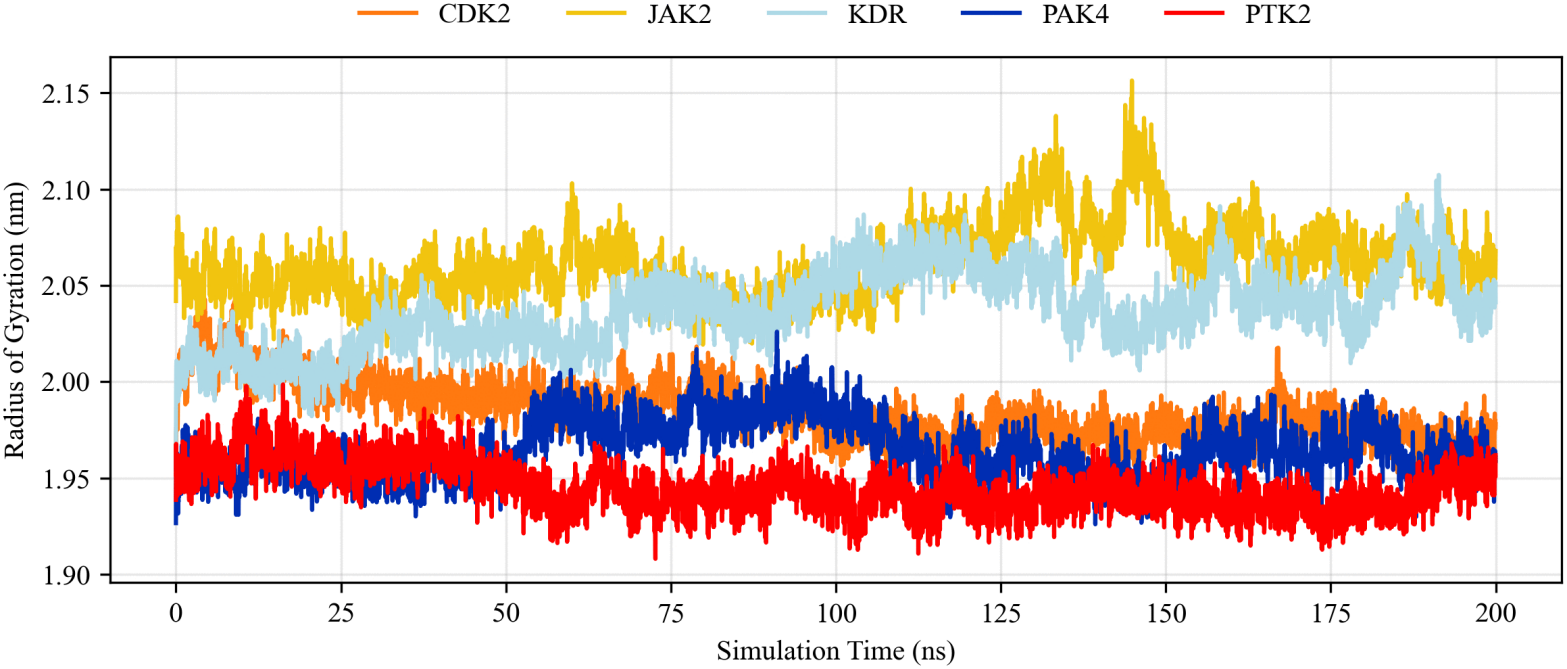
Rg of baicalein complexed with different receptor proteins over the simulation time.

The Rg parameter is used to calculate the size change or tightness of the protein-ligand binding during molecular dynamics simulations [54]. The lower the Rg value, the tighter the protein-ligand complex and the better the system stability. As shown in Fig 6, the Rg values of the baicalein complex are all within the reference range.

**Fig 7:**
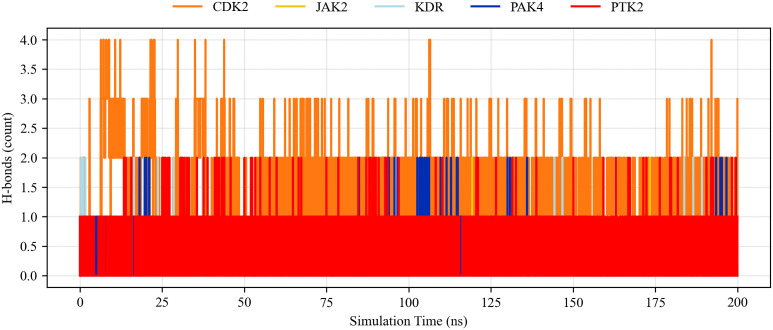
Total number of hydrogen bonds between baicalein complexes with different ligand-proteins.

Fig 7 also shows the total hydrogen bond interactions between each receptor protein and baicalein during the simulation. The calculation results indicate that the number of hydrogen bonds formed by baicalein during the simulation ranged from 0 to 4; therefore, the selected ligands formed a higher number of hydrogen bonds than the internal ligands. Overall, the results of the molecular dynamics simulation are consistent with the results of the molecular docking studies.

**Fig 8:**
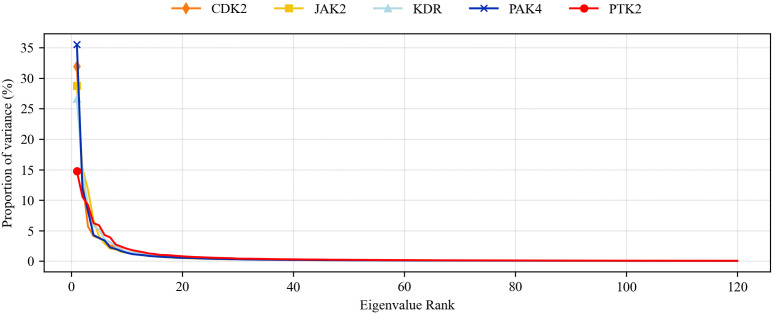
Ranking of characteristic values of baicalein complexes.

**Fig 9:**
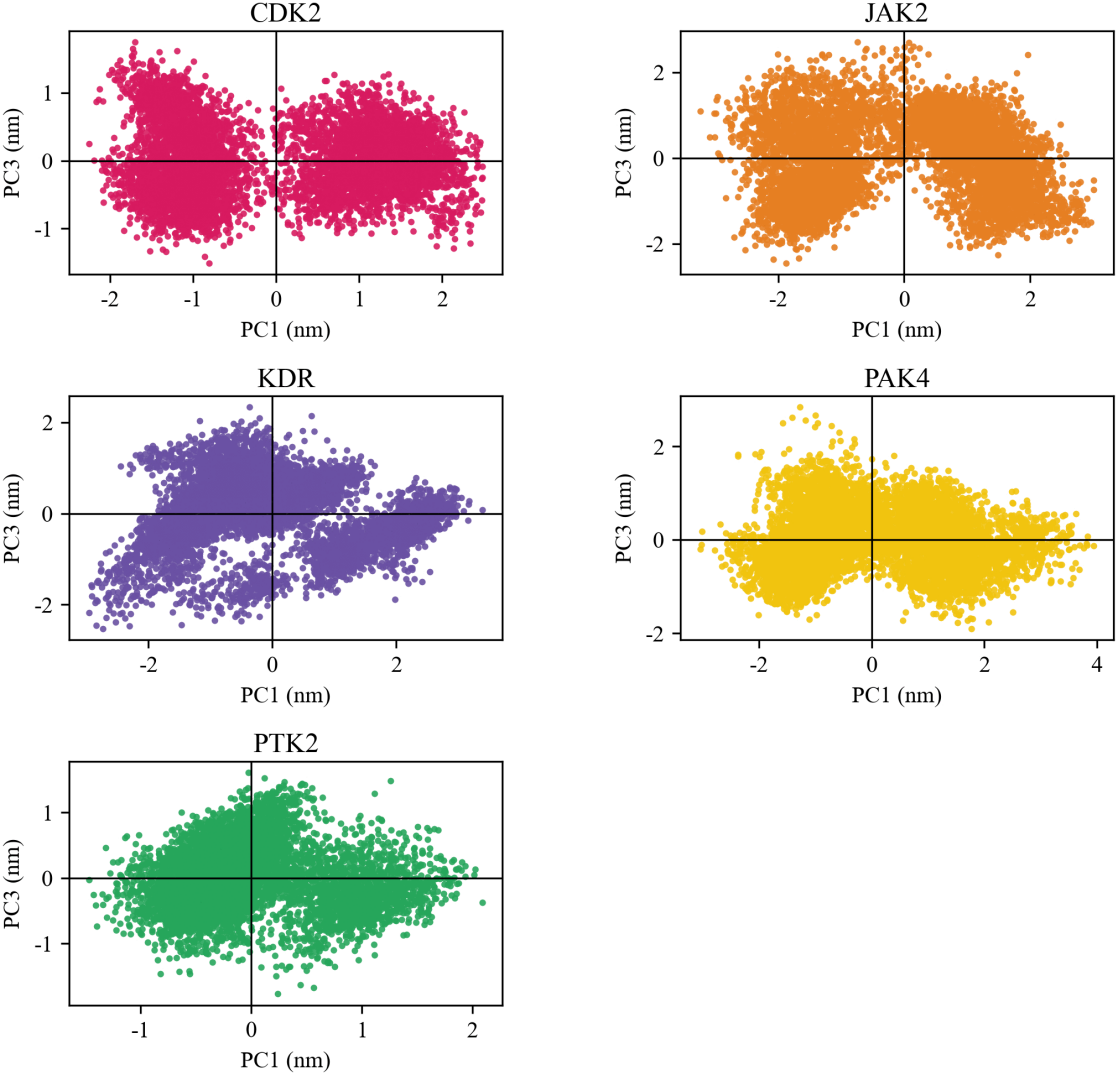
The scree plot for PC1 versus PC3 of baicalein and receptor.

Principal component analysis (PCA), or eigendynamics, is an advanced method in molecular dynamics simulations [55]. For example, biomacromolecules with numerous degrees of freedom, such as proteins, can undergo significant conformational changes, resulting in complex shapes and exhibiting a wide range of activities [56]. PCA analysis helps to study significant coordination motions that occur during ligand binding. In this study, eigenvectors were obtained through matrix diagonalization. Fig 8 shows the eigenvalues obtained by diagonalizing the atomic fluctuation covariance matrix of the baicalein complex, arranged in descending order, corresponding to the respective eigenvectors. Fig 9 shows the two-dimensional projections of the trajectories of the first three principal components, PC1 and PC3, of the baicalein complex in phase space. According to the PCA results, the studied baicalein complex exhibits relatively small motions and maintains stable interactions.

### MM-PBSA binding free energy results

**Fig 10:**
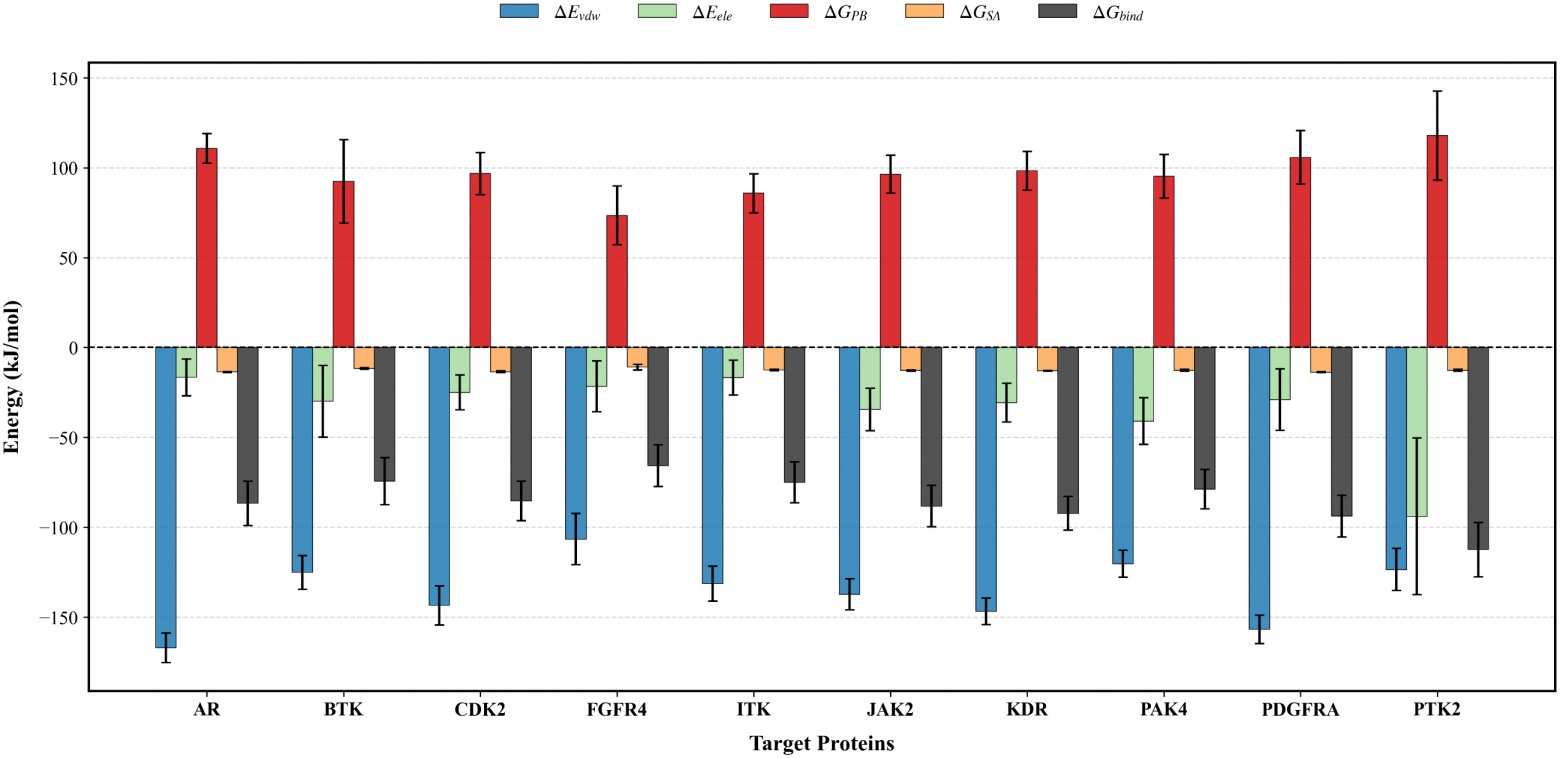
MM/PBSA binding free energy and energy components for different target protein complexes.

Table 1 lists the binding free energies of the natural compound baicalein and its respective protein targets. As shown in Fig 10, the MM/PBSA results show that baicalein exhibits high binding free energies with JAK2 (−88.32 kJ mol^−1^) and PTK2 (−112.51 kJ mol^−1^), while its interactions with BTK and FGFR4 are weaker(S8 Fig and S2 Table). Van der Waals forces contribute significantly to the total free energy, while electrostatic energy contributes less. The binding site is primarily dominated by non-polar hydrophobic interactions, which is consistent with the hydrophobic embedding characteristics observed in the docking results. Solvent accessible surface area (SASA) [57] represents the degree of solvent exposure. The total binding free energy of baicalein suggests that it can effectively bind to lung cancer-related gene targets such as JAK2 and PTK2.

**Table 1:** The MM/PBSA binding free energy of baicalein corresponding proteins (KJ mol^−1^).

Proteins	$\Delta E_{vdw}$	$\Delta E_{ele}$	$\Delta G_{PB}$	$\Delta G_{SA}$	$\Delta SASA/nm^{2}$	$\Delta G_{binding}$
AR	−167.11 ± 8.20	−16.69 ± 10.33	110.75 ± 8.28	−13.72 ± 0.29	−4.56 ± 0.10	−86.73 ± 12.30
BTK	−125.19 ± 9.33	−29.92 ± 19.92	92.34 ± 23.18	−11.72 ± 0.46	−3.89 ± 0.15	−74.48 ± 13.10
CDK2	−143.47 ± 10.8	−25.02 ± 9.62	96.73 ± 11.72	−13.60 ± 0.50	−4.51 ± 0.17	−85.40 ± 10.92
FGFR4	−106.65 ± 14.27	−21.63 ± 14.10	73.43 ± 16.36	−10.96 ± 1.63	−3.64 ± 0.54	−65.86 ± 11.63
ITK	−131.38 ± 9.71	−16.78 ± 9.67	85.69 ± 10.84	−12.59 ± 0.33	−4.18 ± 0.11	−75.06 ± 11.30
JAK2	−137.24 ± 8.66	−34.60 ± 11.84	96.36 ± 10.63	−12.89 ± 0.38	−4.28 ± 0.12	−88.32 ± 11.51
KDR	−146.82 ± 7.36	−30.75 ± 10.71	98.24 ± 10.71	−13.01 ± 0.25	−4.32 ± 0.08	−92.34 ± 9.41
PAK4	−120.42 ± 7.49	−41.00 ± 12.93	95.23 ± 12.18	−12.72 ± 0.46	−4.22 ± 0.15	−78.87 ± 10.92
PDGFRA	−156.77 ± 7.91	−29.04 ± 17.15	105.69 ± 14.90	−13.77 ± 0.29	−4.57 ± 0.10	−93.89 ± 11.67
PTK2	−123.55 ± 11.72	−93.97 ± 43.51	117.78 ± 24.73	−12.72 ± 0.46	−4.22 ± 0.15	−112.51 ± 15.02

### Pharmacokinetic profile

In recent years, researchers have used several techniques to predict the druggability of lead compounds, such as the Lipinski rule (Ro5) and the ADMET parameter [58]. The three parameters of Ro5 include molecular weight, hydrogen bond acceptor, and hydrogen bond donor, which are related to the interaction between the ligand and the active site of the protein. The lipophilicity parameter (Log P), unlike the other three parameters, is independent of the biophysical properties of the target and can be calculated experimentally. Topological polar surface area (TPSA) characterizes the ability of a compound to penetrate cells [59]. When the TPSA value is greater than 140 Å^2^ [60], the compound’s ability to penetrate the cell membrane decreases. Predicting drug distribution in the human body depends on several parameters related to in vivo distribution, namely human intestinal absorption rate (HIA%), in vitro plasma protein binding rate, and blood-brain barrier (BBB) permeability [61].

The physicochemical properties and pharmacokinetic characteristics of the screened natural compounds of Baicalein, namely baicalein, Baicalin, Xanthosine, (-)-beta-Sitosterol, Cavidine, coniferin, Cycloartenol, cis-11-Eicosenoic acid, Stigmasterol, 10,13-Eicosadienoic acid, 12,13-Epoxy-9-hydroxynonadeca-7,10-dienoic acid and 24-Ethylcholest-4-en-3-one, were investigated using Swiss ADME and PreADMET software. As shown in Table 2, baicalein conformed to Lipinski’s rule (violation count = 0) and had few rotatable bonds (1–2), indicating a reasonable basic structure for oral lead compounds. The LogP value (1.71) and TPSA value (90.9 Å²) of baicalein were both within the range favorable for membrane permeability. Baicalein is a small, compact compound with suitable hydrophilic and hydrophobic properties, fitting the “template image” of early oral lead compounds.

**Table 2:** Physical and chemical properties of compounds in Pinellia ternata.

Compounds	*MW*(*g mol*^-1^)	LogP	HBD	HBA	TPSA(*Å*^2^)	n-RB	Lipinski violation
10,13-Eicosadienoic acid	308.5	6.18	1	2	37.3	16	1
12,13-Epoxy-9-hydroxy nonadeca-7,10-dienoic acid	324.45	4.01	2	4	70.06	14	0
24-Ethylcholest-4-en-3-one	412.69	7.36	0	1	17.07	6	1
baicalein	270.24	1.71	3	5	90.9	1	0
Baicalin	444.35	0.07	4	11	192.78	4	1
Xanthosine	284.23	−1.85	5	7	153.46	2	0
(-)-beta-Sitosterol	414.71	7.24	1	1	20.23	6	1
Cavidine	353.41	3.23	0	5	40.16	2	0
coniferin	342.34	−0.48	5	8	128.84	6	0
Cycloartenol	426.72	7.51	1	1	20.23	4	1
cis-11-Eicosenoic acid	310.51	6.41	1	2	37.3	17	1
Stigmasterol	412.69	6.98	1	1	20.23	5	1

As shown in Table 3, baicalein exhibits a high intestinal absorption rate (HIA) (88.1%), but is significantly weak in the human Caco-2 permeability model (12.8 nm·s^−1^) and low skin permeability (0.26 cm h^−1^). Regarding distribution, baicalein has extremely high plasma protein binding (PPB ≈ 99% − 97.8%) and very low free fraction. Blood-brain barrier prediction indicates that baicalein (0.77%) more readily enters the central nervous system, which is significant for peripheral administration to tumors and represents a distribution pattern that is relatively “peripherally safe”.

**Table 3:** In silico ADME analysis of compounds in Pinellia ternata.

Entry	Absorption		Distribution		
compounds	%HIA	In vitro Caco-2 cell permeability (nm s^−1^)	In vitro skin permeability (lookup, ${cm\;h}^{-1}$)	% in vitro plasma protein binding	%BBB
10,13-Eicosadienoic acid	98.2089	304.198	1103.6885	100	10.56
12,13-Epoxy-9-hydroxy nonadeca-7,10-dienoic acid	95.2753	225.507	430.5162	100	1.4158
24-Ethylcholest-4-en-3-one	100	538.178	929.8427	100	19.559
baicalein	88.105491	12.8026	0.2635	98.983295	0.7708
Baicalin	25.3336	5.7073	0.1207	51.7322	0.015
Xanthosine	24.7163	136.448	0.0215	12.7683	0.291
(-)-beta-Sitosterol	100	523.734	918.044	100	19.888
Cavidine	97.7466	560.997	0.2723	81.8264	0.0544
coniferin	52.107	141.527	0.1219	37.6157	0.0456
Cycloartenol	100	500.256	34.7922	100	20.37
cis-11-Eicosenoic acid	98.3144	305.319	1099.8501	100	12.743
Stigmasterol	100	523.376	689.6607	100	19.894

Table 4 lists the toxicity analysis results of natural compounds in Pinellia ternata. Baicalein showed Ames positivity and a moderate risk of hERG inhibition, but did not exhibit carcinogenicity in mice; however, these two points need to be addressed in structural optimization and further validation. In the algal acute toxicity score, baicalin scored 0.051, suggesting that the environmental toxicological burden of coniferin may be relatively low.

**Table 4:** Toxicity analysis of compounds in Pinellia ternata.

Entry	algae_at	Ames_test	Carcino_Mouse	hERG_inhibition
10,13-Eicosadienoic acid	0.001	non-mutagen	positive	low_risk
12,13-Epoxy-9-hydroxy nonadeca-7,10-dienoic acid	0.007	mutagen	positive	low_risk
24-Ethylcholest-4-en-3-one	0.001	non-mutagen	positive	low_risk
baicalein	0.051	mutagen	negative	medium_risk
Baicalin	0.066	mutagen	positive	ambiguous
Xanthosine	0.354	mutagen	negative	medium_risk
(-)-beta-Sitosterol	0.001	non-mutagen	positive	low_risk
Cavidine	0.025	mutagen	negative	low_risk
coniferin	0.081	mutagen	negative	medium_risk
Cycloartenol	0.001	non-mutagen	positive	low_risk
cis-11-Eicosenoic acid	0.001	non-mutagen	negative	low_risk
Stigmasterol	0.001	non-mutagen	positive	low_risk

### DFT analysis

**Fig 11:**
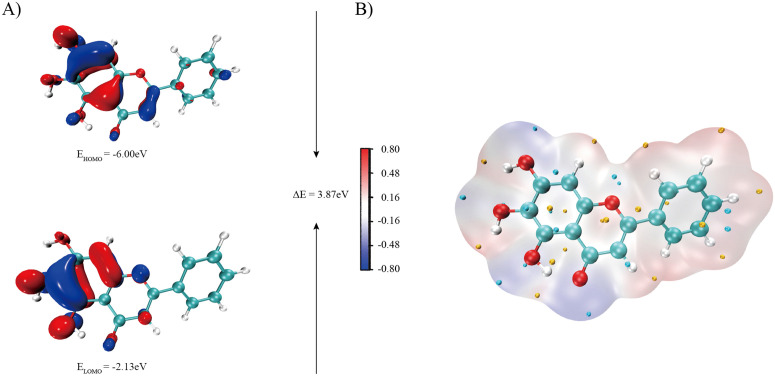
A) DFT calculated LUMO, HOMO, and their energies for baicalein at the B3LYP-D3/def2-TZVP level of theory. B) ESP of the corresponding conformations of baicalein at B3LYP-D3/def2-TZVP level of theory.

DFT calculations further revealed the intrinsic properties of the compounds’ reactivity and interactions [62,63]. Frequency and optimization calculations were performed on baicalein, Baicalin, Xanthosine, (-)-beta-Sitosterol, Cavidine, coniferin, Cycloartenol, cis-11-Eicosenoic acid, Stigmasterol, 10,13-Eicosadienoic acid, 12,13-Epoxy-9-hydroxynonadeca-7,10-dienoic acid and 24-Ethylcholest-4-en-3-one using the B3LYP-D3/def2-TZVP method, yielding the total electron density (Etot), electron affinity A, hardness η, and softness σ (as shown in Table 5). The HOMO and LUMO molecular orbitals were used to predict the reactivity and physical and structural properties of the compounds. The HOMO and LUMO energies and the energy difference between the HOMO and LUMO are shown in Fig 11. The band gap energy of baicalein is 3.87 eV. This indicates that baicalein exhibits higher chemical reactivity than other active components of Pinellia ternata due to its lower band gap between the HOMO and LUMO(S3 Fig).

**Table 5:** The calculated total energy (Etot), Enthalpy (H), Gibbs free energy (G), hardness (η), softness (σ, and electron affinity (A) of compound from Pinellia ternata at B3LYP/6-31 + G(d,p) level of theory [^*a*^in Hartree/ particle, ^*b*^in cal/mol K, ^*c*^in ev, ^*d*^in ev^−1^].

Compounds	${𝐄}_{tot}^{a}$	${𝐇}^{a}$	${𝐆}^{a}$	${𝐒}^{b}$	$\eta^{c}$	$\sigma^{d}$	${𝐀}^{c}$
10,13-Eicosadienoic acid	−934.091	−933.537	−933.623	182.19	3.289	0.304	−0.02
12,13-Epoxy-9-hydroxy nonadeca-7,10-dienoic acid	−1043.986	−1043.474	−1043.560	181.61	3.096	0.322	0.70
24-Ethylcholest-4-en-3-one	−1209.001	−1208.250	−1208.339	186.18	2.555	0.391	1.33
baicalein	−953.675	−953.437	−953.495	121.85	1.94	0.52	2.13
Baicalin	−1638.436	−1638.032	−1638.115	174.47	1.813	0.551	2.20
Xanthosine	−1058.590	−1058.328	−1058.390	131.20	2.796	0.357	0.64
(-)-beta-Sitosterol	−1210.196	−1209.422	−1209.51	188.83	3.403	0.293	−0.47
Cavidine	−1169.536	−1169.107	−1169.180	152.70	2.622	0.381	0.3
coniferin	−1224.57	−1224.172	−1224.249	163.01	2.376	0.420	1.14
Cycloartenol	−1248.253	−1247.473	−1247.564	189.93	3.418	0.292	−0.55
cis-11-Eicosenoic acid	−935.317	−934.738	−934.826	184.13	3.307	0.302	−0.06
Stigmasterol	−1208.969	−1208.220	−1208.308	187.01	3.356	0.297	−0.42

The electronic responses were analyzed using frontier orbitals and electrostatic potentials (ESPs), as shown in Fig 11. The red spheres on the ESP plots represent negatively charged sites. The ESP spectra specify the equilibrium charge distribution in baiaclein, which is beneficial for the compound’s binding to biological enzymes. Baicalein’s ground-state energy and thermodynamic free energy are in a relatively stable range, with low conformational entropy and a relatively compact spatial configuration. Among natural Pinellia ternata compounds, it belongs to a class of compounds with high softness and strong electron affinity(S4 Fig). This explains the highly polarizable and easily stable ligand-forming properties of baicalein as demonstrated in the molecular docking and kinetic simulations described earlier.

The present study is computational and does not include in vitro or in vivo experimental validation of the predicted compound-target interactions and downstream biological effects. Accordingly, our results should be interpreted as providing a prioritized set of candidate mechanisms rather than definitive evidence of efficacy or causality. Future studies are warranted to substantiate these predictions using appropriate experimental models, for instance, binding and enzymatic assays for the proposed targets, cellular assays for pathway-level effects, and dose-response evaluations, and to assess their pharmacological feasibility and potential off-target effects.

## Conclusion

This study constructs a hierarchical integrated computational evidence chain which named CrossScale-Herb to employ comprehensive computational methods to systematically evaluate the potential interactions between natural compounds in Pinellia ternata and lung cancer-related targets. Through using the HERBGAT network, we identified protein targets with cross-targeting effects on lung cancer and reveal the multi-target mechanisms of these natural compounds. By integrating evidence from PPI topological hubs, docking confidence, dynamic stability, and energy decomposition, we identified a highly correlated migration-angiogenesis-cell cycle node axis centered on PTK2, KDR, JAK2, and CDK2.

Among these compounds, baicalein exhibited consistent mechanistic characteristics across targets such as PTK2/KDR/JAK2. Besides, molecular dynamics simulations and binding free energy calculations showed that baicalein forms stable complexes with lung cancer-related targets, considering binding energy values consistently falling within the ideal range. These complexes are core-driven by hydrophobic interactions and anchored by local polar interactions, while a gatekeeper aromatic layer provides a selective entry site. This complex possesses cross-targeting, transferable pharmacophore features, facilitating targeted optimization in the side pocket dimension. ADMET property predictions further confirmed that although all three compounds comply with drug-likeness rules and have the potential as oral lead compounds, baicalein requires structural optimization to address Ames test and hERG risks, while improvements in its transmembrane permeability and excessive protein binding are needed. This provides strong computational evidence for the anti-cancer mechanisms of natural compounds in Pinellia ternata. DFT analysis revealed high electronic softness, frontier orbital distribution, and electrostatic potential responses that highly correlate with ligand-receptor electronic matching in multi-target cavities, providing electronic corroboration for the energy and conformational study results.

Baicalein can serve as a preferred chemical starting point for experimental validation of lead compound optimization and combination intervention hypotheses centered on tumor-related nodes such as PTK2/FGFR4/CDK2/PDGFRA/AR. This can be extended to other ethnomedicinal resources and diseases, thereby accelerating the discovery of innovative therapies inspired by traditional medicine.

## Supporting information

S1 File Supplementary method description. (DOCX)

S1 Fig Selected natural compounds of Pinellia ternate. (TIF)

S2 Fig Interaction and orientation of baicalein in A) AR, B) BTK, C) FGFR4, D) ITK, E) PAK4 and F) PDGFRA. (TIF)

S3 Fig DFT calculated LUMO, HOMO, and their energies for compounds of Pinellia ternata at the B3LYP-D3/def2-TZVP level of theory. (TIF)

S4 Fig ESP of the corresponding conformations of compounds of Pinellia ternata at B3LYP-D3/def2-TZVP level of theory. (TIF)

S5 Fig PPI network of all identified genes. (TIF)

S6 Fig Network of all the metabolites of Pinellia ternata, their targets and signalling pathways. (TIF)

S7 Fig Hub targets identified by using Cytohubba plugin of the Cytoscape software. (TIF)

S8 Fig MM/GBSA binding free energy and energy components for different target protein complexes. (TIF)

S1 Table Ranking of candidate targets based on the weighted confidence index. (XLSX)

S2 Table The MM/GBSA binding free energy of baicalein corresponding proteins (KJ mol^-1^). (XLSX)
