# Supplementary material for: In-silico prediction of multi‑target mechanisms of Pinellia ternata phytochemicals in lung cancer: Evidence from a graph‑attention‑guided virtual screening and multi‑scale simulations
Source: PLoS One. 2026 May 18;21(5):e0349376. doi: 10.1371/journal.pone.0349376 (PMC13183200; doi:10.1371/journal.pone.0349376)
Supplement: S1 File — (DOCX) [file pone.0349376.s001.docx]

Supplementary Material

# Supplementary method description

In order to identify the most potential drug targets from the large-scale molecular docking simulation, we constructed and implemented a hierarchical consensus based post-processing and sequencing process. This process aims to go beyond the single optimal docking score, and give priority to the evaluation of the stability and reproducibility of the prediction combination mode by integrating the statistical characteristics of multiple independent simulations. The core of the analysis process is a composite receptor confidence index. Its construction process includes two main sections:

Confidence evaluation of pair level: for each unique receptor ligand pair, we firstly aggregate its data in all repeated runs to calculate one pair confidence index which is a weighted linear combination, and its composition is as follows: **1.** Fraction of High-Confidence Poses (60%): this indicator can quantify the frequency of high confidence combination patterns in multiple docking. As a key surrogate variable for predicting the conformational stability of binding modes, it is given the highest weight, reflecting our "stability first" screening philosophy. **2.** Normalized Median Docking Score (25%): to suppress the statistical deviation caused by a single extreme high score, we use the median of all operation scores and normalize them by robust scaling based on the 5th and 95th percentiles. This will reflect the typical binding affinity level of the pair. **3.** Normalized Best Docking Score (15%): this item retains the consideration of the potential highest affinity, but the weight is low to avoid leading the assessment with false positive results.

Potential aggregation of receptor level: after obtaining the confidence of each pair, we aggregate the data to each independent receptor to calculate its overall potential as a drug target, namely receptor_conf_index. The index consists of two equal weight parts: **1.** Mean Fraction of High-Confidence Poses (50%): it measures the general tendency of a receptor to form a stable binding mode with a series of different ligands, reflecting its potential "drug resistance" or universality to small molecular ligands. **2.** Mean Confidence Index of Top-3 Pairs (50%), focuses on the binding quality of the receptor with several ligands with the best performance, representing the upper limit of its potential for binding with the optimized lead compound.

Finally, in order to correlate our computational predictions with established biological knowledge, we introduce a literature informed boost step. For those receptors that have been identified as key disease targets (e.g. KDR, JAK2 and other kinases) in the existing literature, the final receptor_conf_index will be multiplied by a small enhancement factor to obtain the final weighted confidence index. The final target ranking is mainly based on this weighted index, supplemented by secondary indicators such as the number of high confidence leading pairs, to ensure the scientificity and practicability of the recommendation list.
