# Supplementary figures and images for: In-silico prediction of multi‑target mechanisms of Pinellia ternata phytochemicals in lung cancer: Evidence from a graph‑attention‑guided virtual screening and multi‑scale simulations
Source: PLoS One. 2026 May 18;21(5):e0349376. doi: 10.1371/journal.pone.0349376 (PMC13183200; doi:10.1371/journal.pone.0349376)

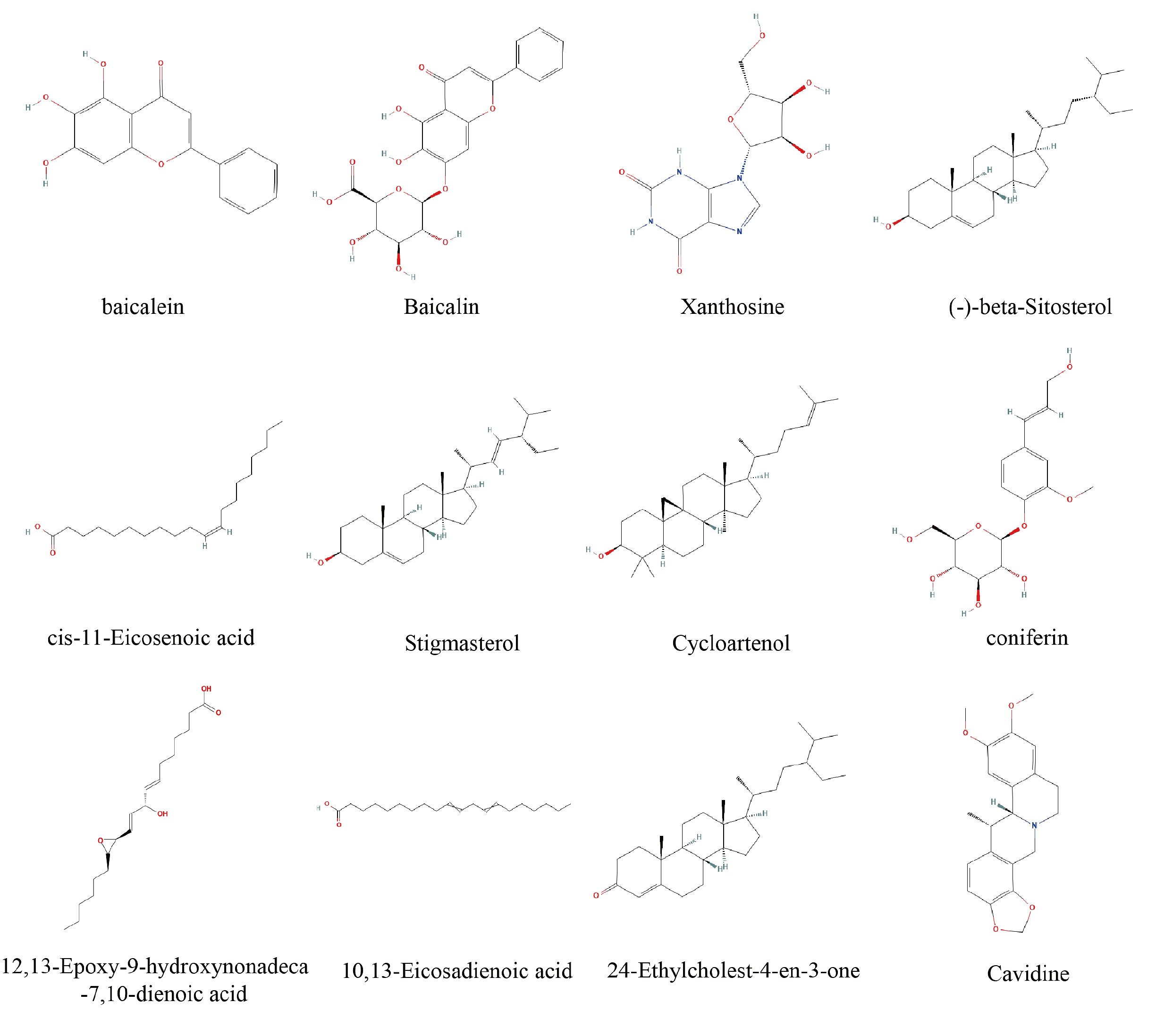

Supplement: S1 Fig — (TIF) [file pone.0349376.s002.tif]

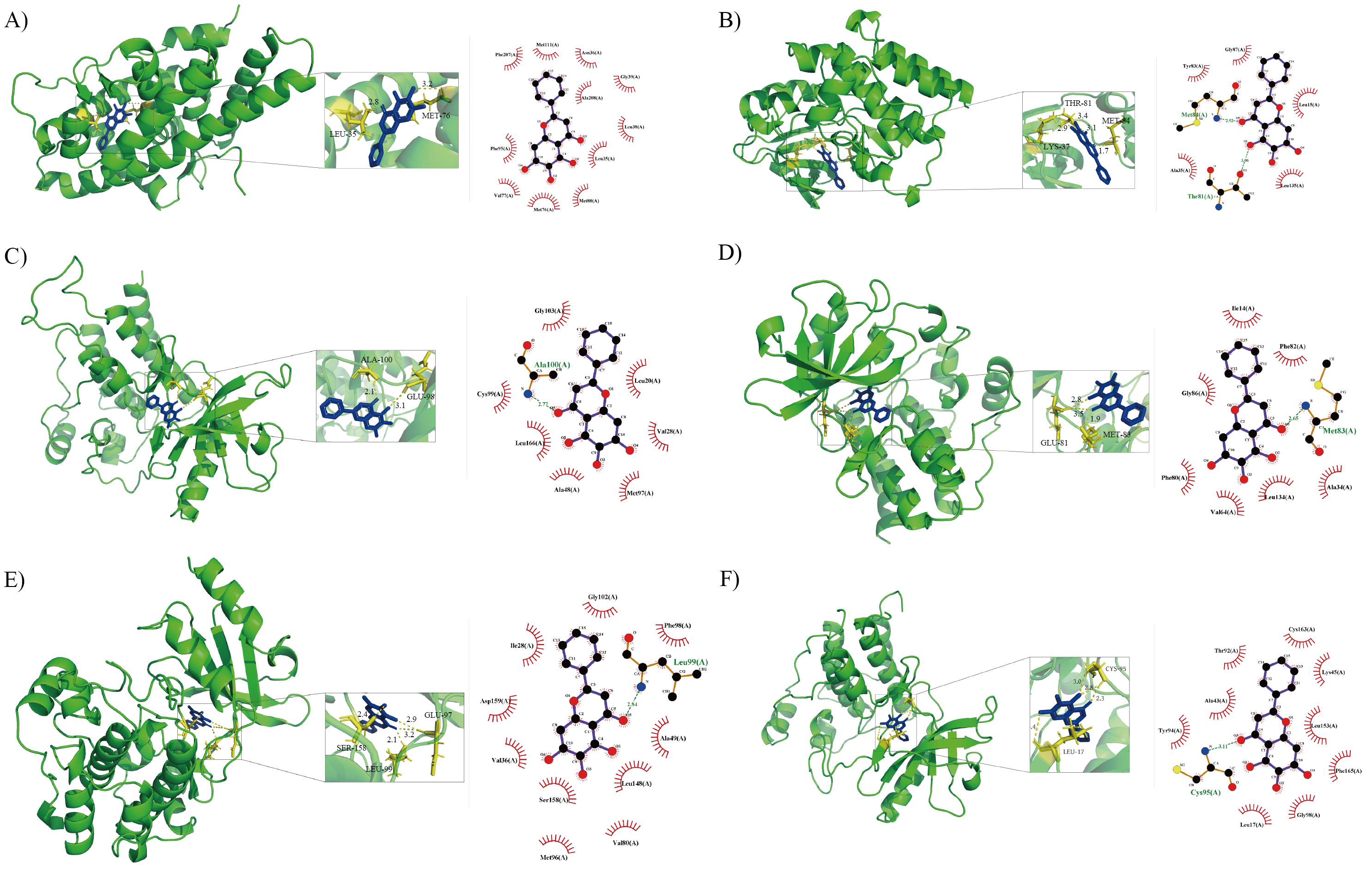

Supplement: S2 Fig — (TIF) [file pone.0349376.s003.tif]

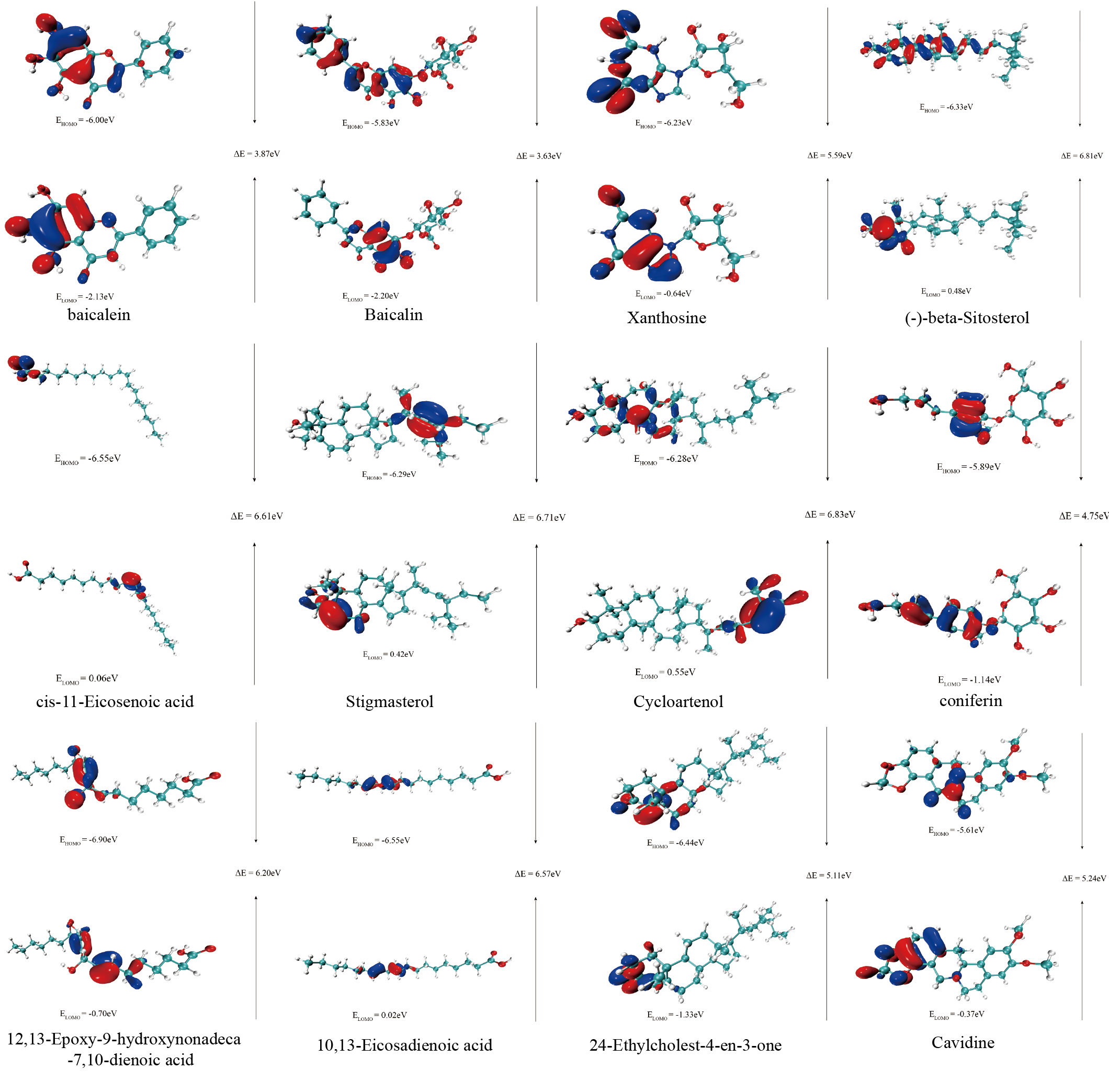

Supplement: S3 Fig — (TIF) [file pone.0349376.s004.tif]

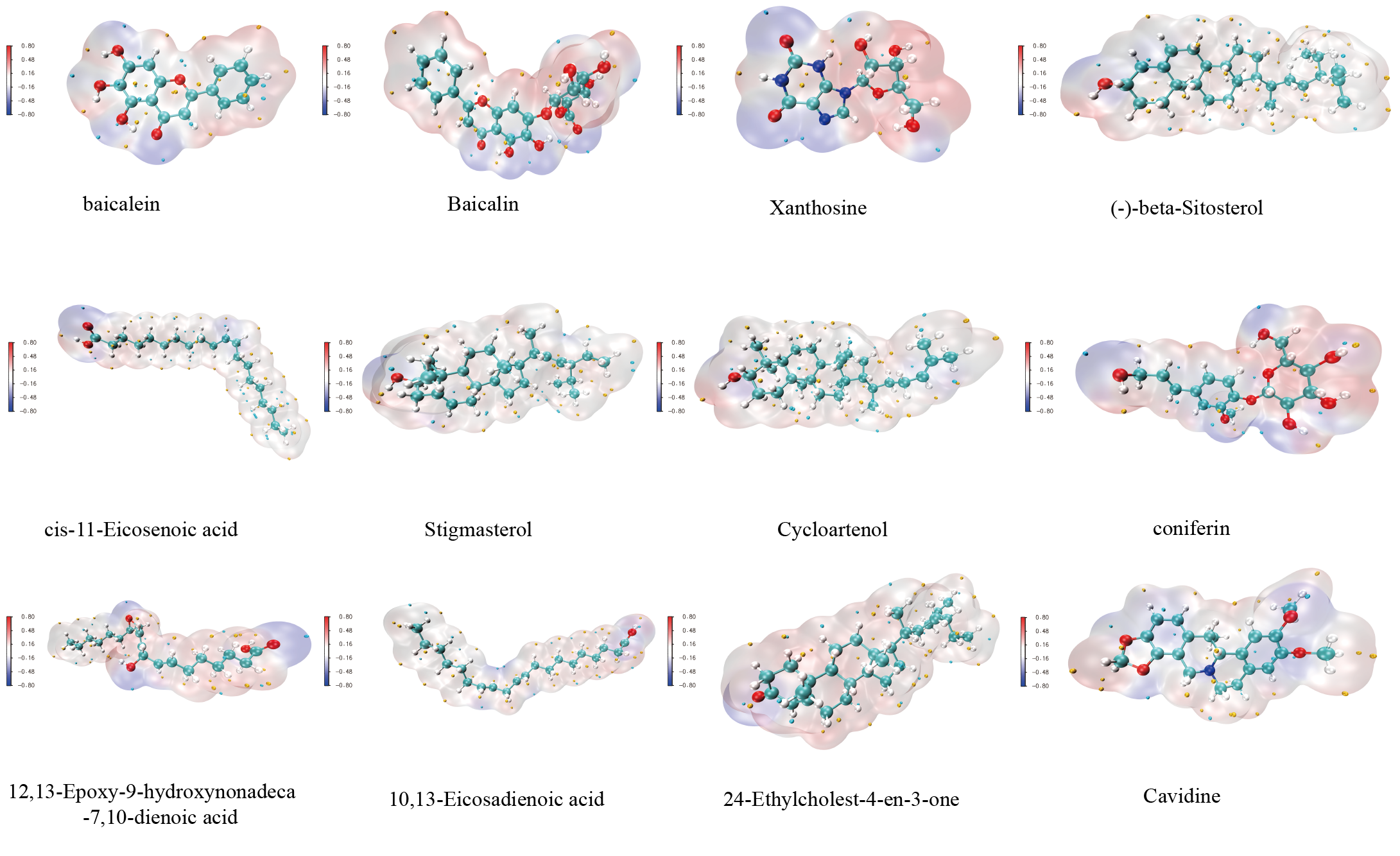

Supplement: S4 Fig — (TIF) [file pone.0349376.s005.tif]

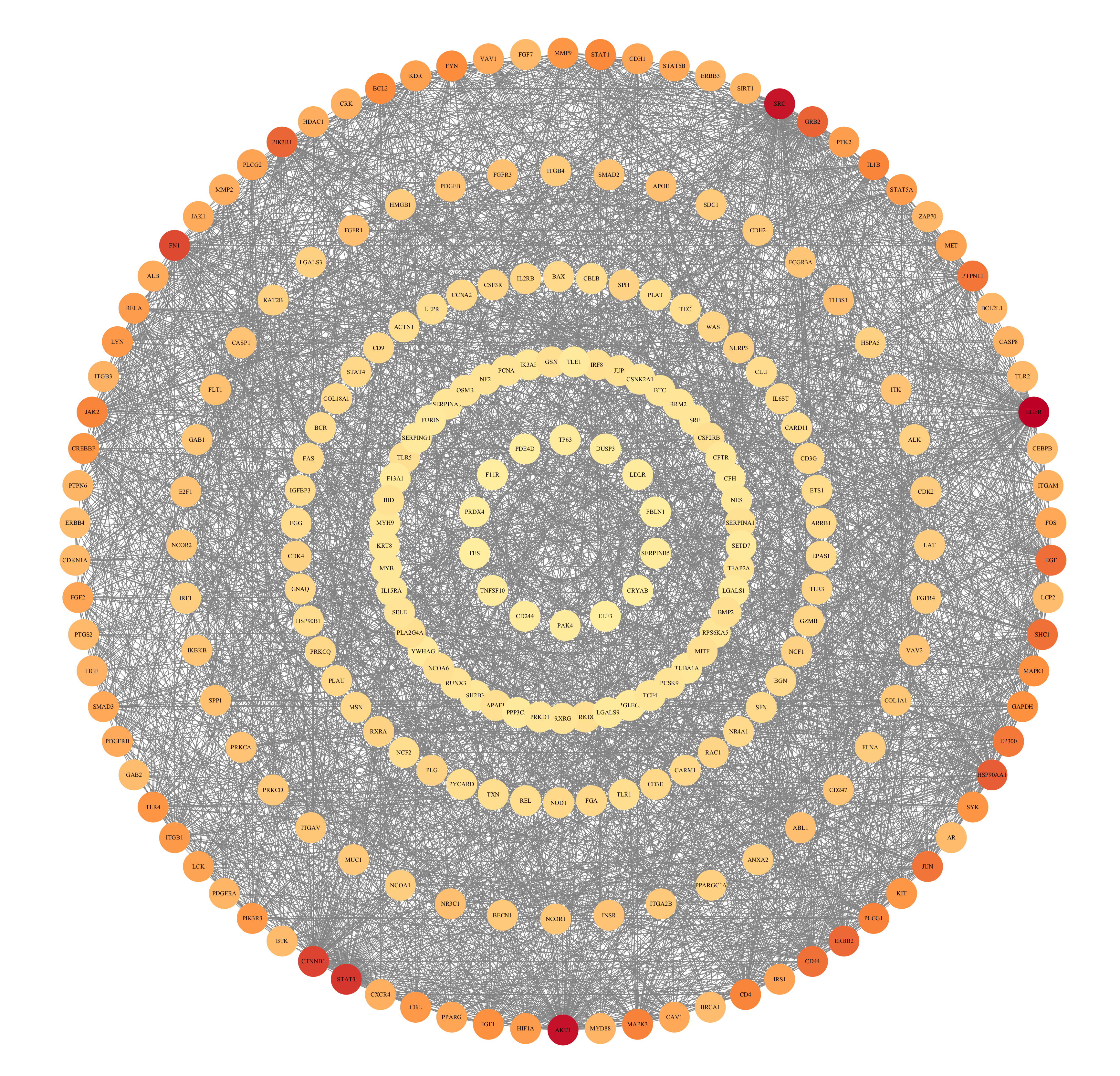

Supplement: S5 Fig — (TIF) [file pone.0349376.s006.tif]

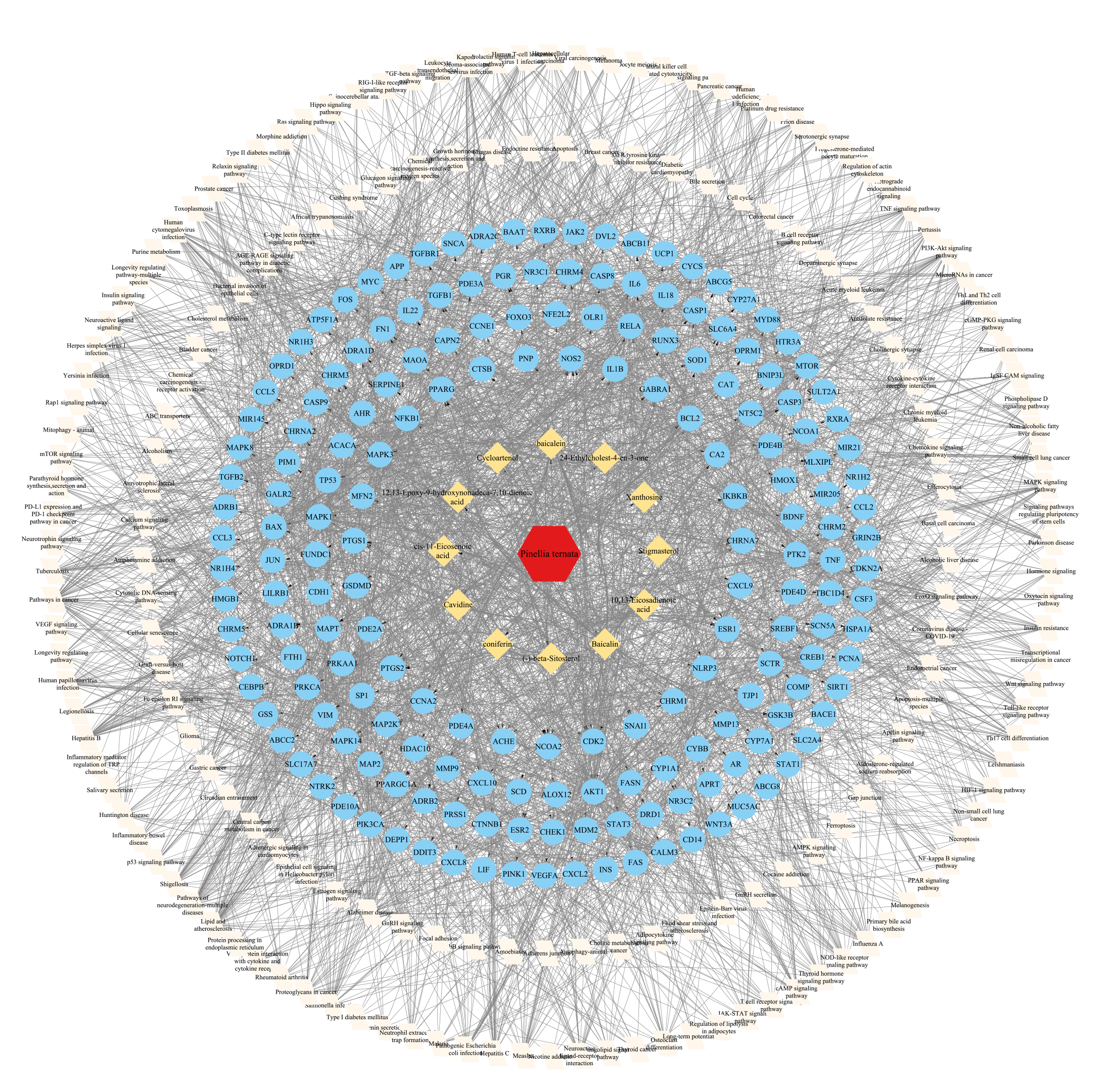

Supplement: S6 Fig — (TIF) [file pone.0349376.s007.tif]

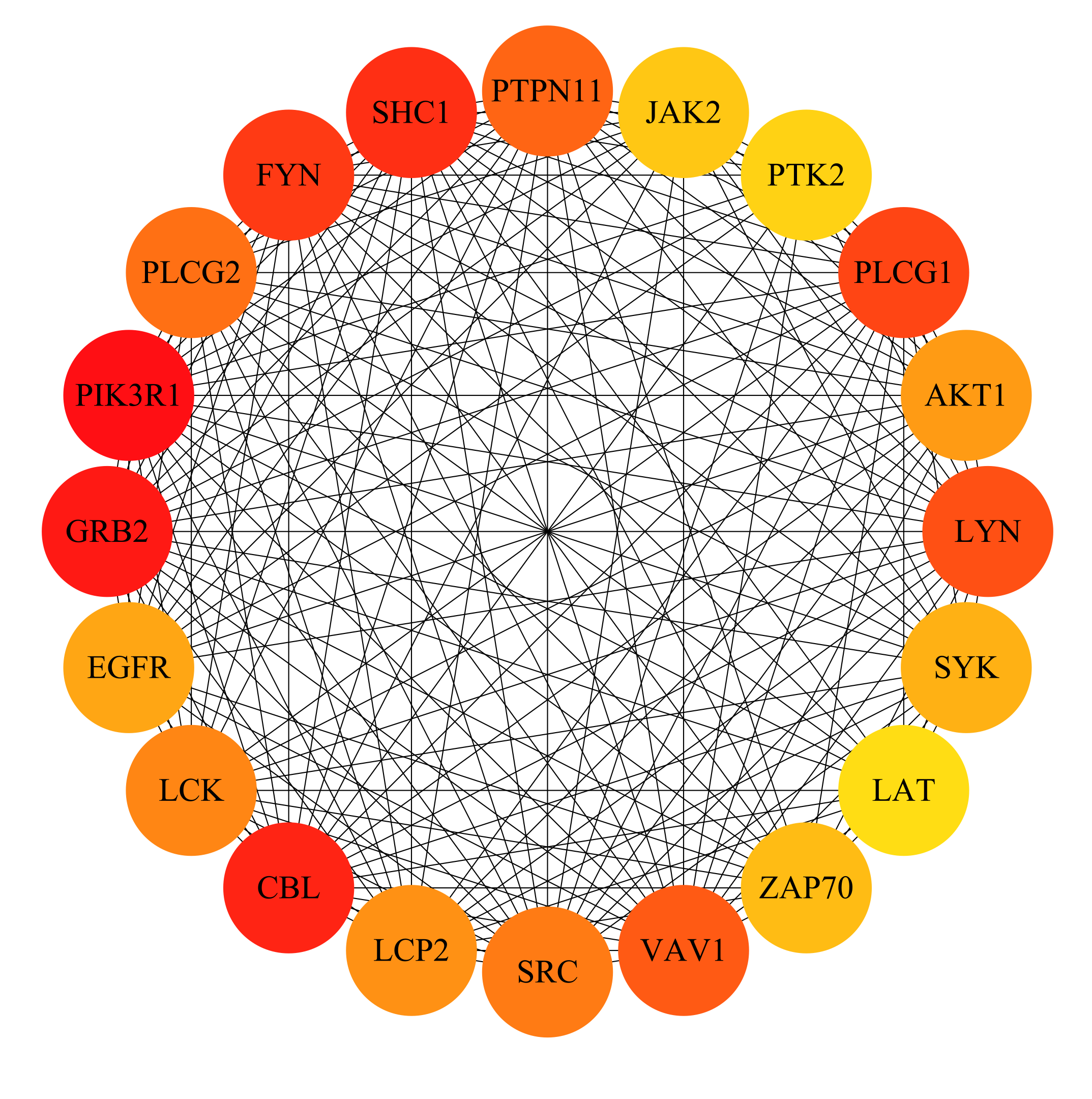

Supplement: S7 Fig — (TIF) [file pone.0349376.s008.tif]

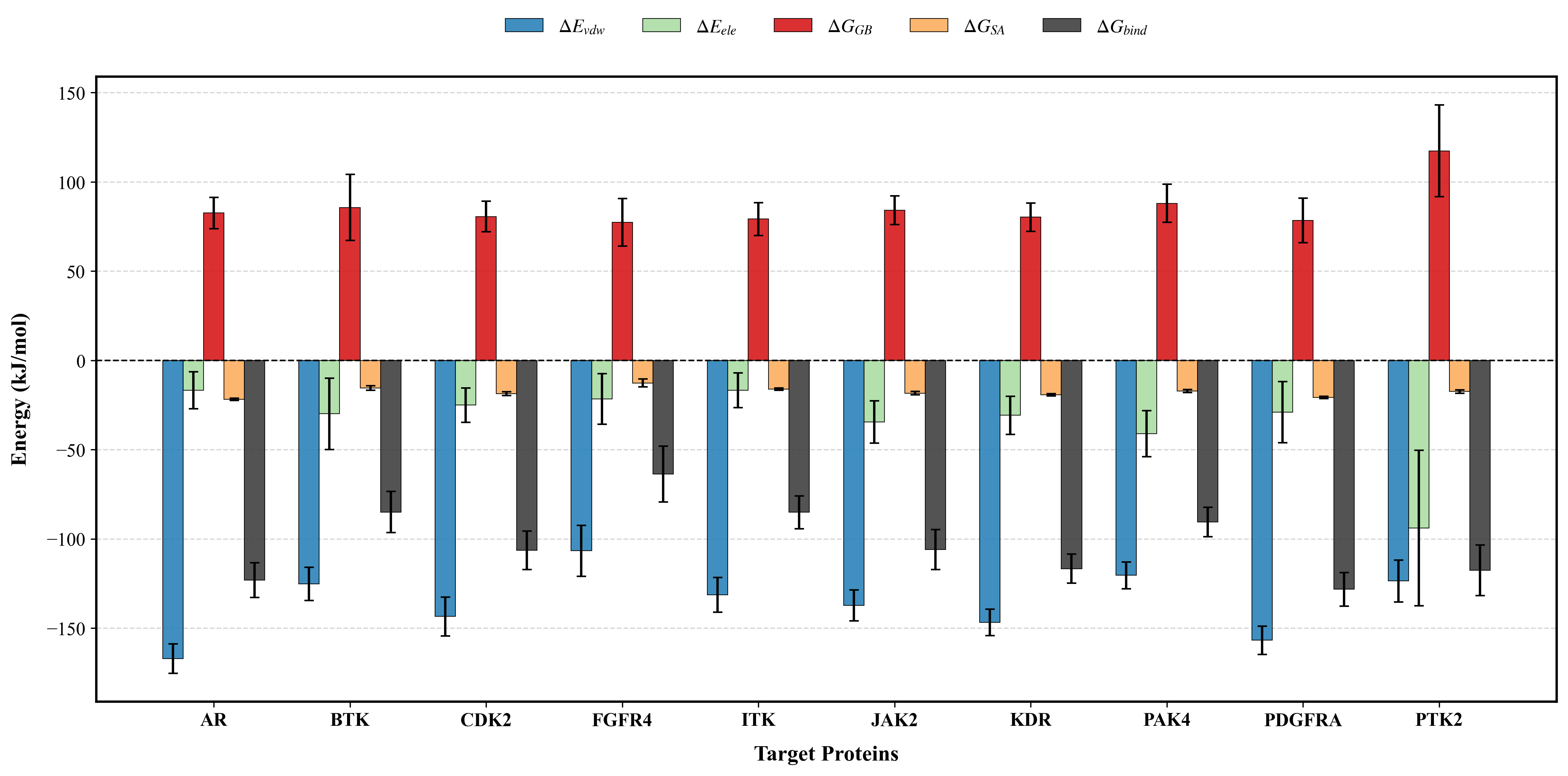

Supplement: S8 Fig — (TIF) [file pone.0349376.s009.tif]
